# Bright Light Emission from Deep Energy States

**DOI:** 10.1002/advs.202509549

**Published:** 2025-08-12

**Authors:** Lian Xiao, Sihang Liu, Zhan Yu, Yong Yi, Rui Duan, Quanchao Du, Yugang Zhao, Xuehong Zhou, Van Duong Ta, Edwin Kok Lee Yeow, Yi Huang, Zhi‐Gang Zheng, Handong Sun

**Affiliations:** ^1^ School of Physics East China University of Science and Technology Shanghai 200237 China; ^2^ Division of Physics and Applied Physics School of Physical and Mathematical Sciences Nanyang Technological University 21 Nanyang Link Singapore 637371 Singapore; ^3^ Research Institute of Aero‐Engine Beihang University No.37 XueYuan Road, Haidian District Beijing 100083 China; ^4^ Beijing An Zhen Hospital Affiliated of Capital University of Medical Sciences Beijing 100029 China; ^5^ Center of Growth Metabolism and Aging Key Laboratory of Bio‐Resource and Eco‐Environment of Ministry of Education College of Life Sciences Sichuan University Chengdu 610064 China; ^6^ Institute of Applied Physics and Materials Engineering University of Macau Macao SAR 999078 China; ^7^ School of Chemistry Chemical Engineering & Biotechnology Nanyang Technological University Singapore 637371 Singapore; ^8^ Shanghai Key Laboratory of Multiphase Flow and Heat Transfer in Power Engineering School of Energy and Power Engineering University of Shanghai for Science and Technology Shanghai 200093 China; ^9^ Department of Optical Devices Le Quy Don Technical University Hanoi 100000 Vietnam

**Keywords:** bright light emission, deep energy states, density of states, sulfur quantum dots

## Abstract

Deep energy states, a fundamental concept in semiconductor physics, play a pivotal role in determining the optical performance of semiconductor materials. They are generally regarded as undesirable defects, as they can serve as efficient centers for non‐radiative recombination. Thus, the prevailing consensus is that deep energy states must be passivated and mitigated to enhance the optical properties of semiconductors. In this work, however, it is demonstrated that these “ undesirable” deep energy states can be harnessed as “ desirable” states to enable bright light emission. This is achieved by proposing a deep energy state regulation mechanism, termed “surface ionization annealing”, which transforms the randomly and broadly distributed non‐radiative deep energy levels into a narrower energy range. This transformation results in a high density of states and band‐edge‐like absorption originating from deep energy states in sulfur quantum dots. The regulation is realized through precise control of surface charge and surface dangling bonds. Simultaneously, surface ionization annealing eliminates non‐radiative deep energy states, significantly enhancing the photoluminescence quantum yield (PLQY) of sulfur quantum dots to 15.6%. Consequently, the simultaneous realization of a high density of states and high PLQY enables bright light emission from deep energy states.

## Graphical abstract

This study proposes a new deep energy state regulation mechanism—surface ionization annealing—and demonstrates that deep energy states can contribute to bright light emission. By applying this mechanism, red light emission from sulfur quantum dots (S‐dots) is achieved.

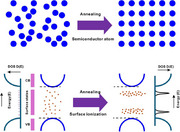

## 1 Introduction

In semiconductors, deep energy states^[^ ^1^ ^]^ refer to energy levels within the bandgap that are situated far from both the conduction band minimum and the valence band maximum, typically more than 0.1–0.3 eV away from either band edge. These states play a critical role in determining the electrical and optical performance of semiconductor materials. Due to their localized nature and low density of states, deep energy levels are generally regarded as undesirable defects,^[^ ^2^ ^]^ acting as efficient centers for carrier trapping and non‐radiative recombination. Their presence often leads to detrimental effects^[^ ^3^ ^]^ such as reduced carrier lifetimes, lower photoluminescence quantum yields, and degraded device (e.g., LEDs^[^ ^4^ ^]^ and photovoltaics^[^ ^5^ ^]^) efficiencies. Consequently, the prevailing consensus^[^ ^6^ ^]^ is that deep energy states should be passivated and mitigated to improve the optical performance of nanostructured semiconductors.

In this work, we demonstrate that deep energy states, traditionally regarded as “undesired,” can also be harnessed as “desired” states to enable bright light emission. It is well recognized that deep energy states are typically randomly distributed within the bandgap, characterized by their localized nature, broad energy distribution, and low density of states, features that are commonly associated with nonradiative recombination. Inspired by annealing manipulation in semiconductors, which facilitates the transformation of randomly distributed atoms into a well‐ordered structure to minimize structural defects and obtain better optoelectronic performance, we propose a similar approach at the electronic level for deep energy states, termed “surface ionization annealing”. In this approach, we first design and introduce deep energy states on the surface of sulfur quantum dots (S‐dots) by incorporating nitrogen atoms into the interstitial sites of the surface S_8_ ring structure. We then regulate the ionization process of these deep energy level states by engineering the surface charge and surface dangling bonds of the S‐dots via ethanol. This process effectively converts the randomly and broadly distributed nonradiative deep energy level states into a narrower energy range, resulting in a high density of states and band‐edge‐like absorption. At the same time, surface ionization annealing leads to the elimination of nonradiative deep energy level states, thus significantly enhancing the photoluminescence quantum yield to 15.6%. Consequently, the combination of a high density of states and improved PLQY enables bright red light emission from deep energy states. Our results offer new insights and an alternative approach for achieving bright light emissions from semiconductors.

## 2 Results and Discussion

### 2.1 Deep Energy States Generation Within the Band Gap of Sulfur Quantum Dots

**Figure 1:**
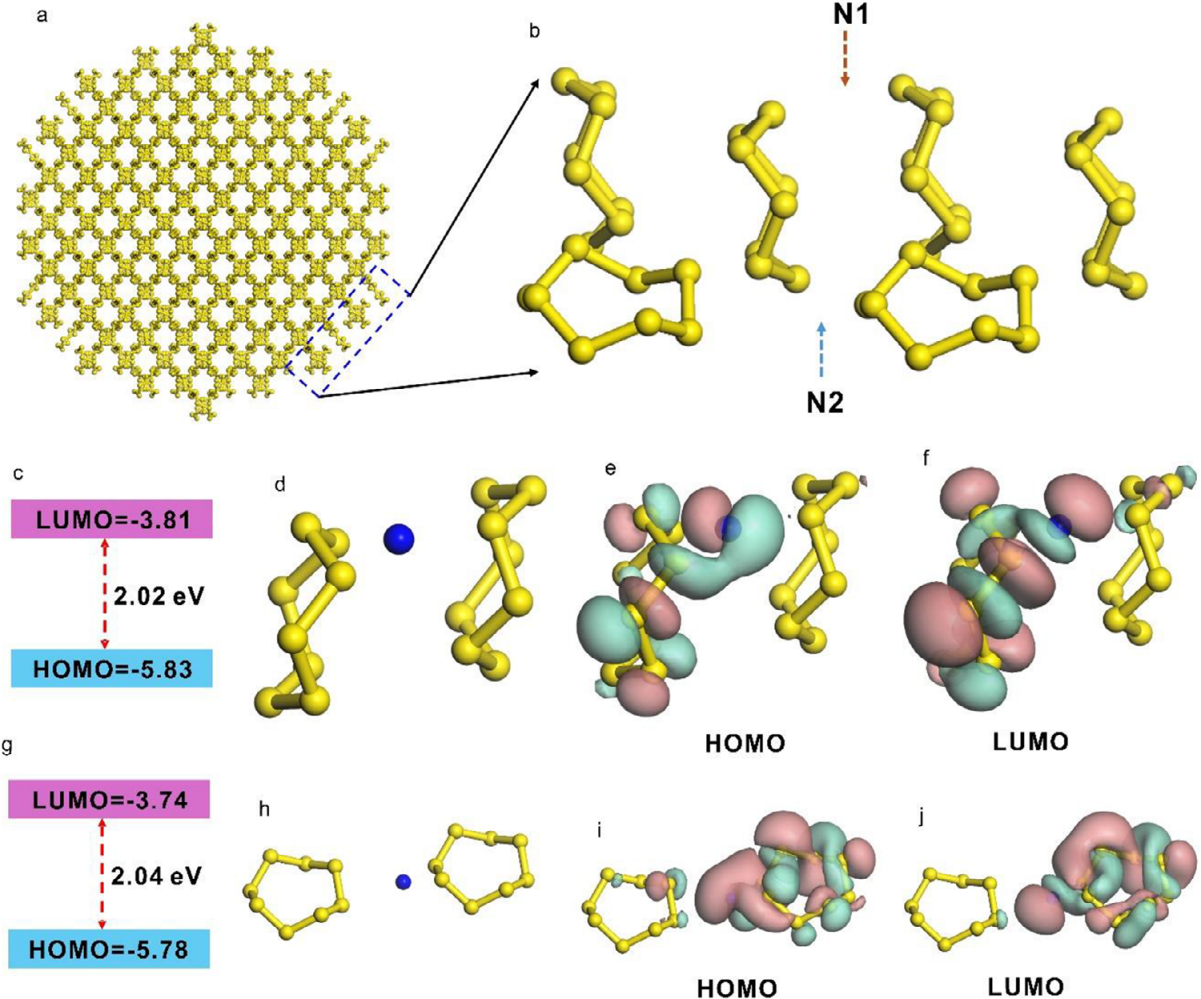
a,b) Illustration of the nitrogen atom insertion between two S_8_ rings. Structure N1: nitrogen atom inserted between two vertical S₈ ring structures. N2: nitrogen atom inserted between two horizontal S₈ ring structures. c–f): Theoretical calculation results for N1 nitrogen atom structure. (c) Calculated LUMO and HOMO energy for structure (d). (d) localized structure when a N1 nitrogen atom is inserted between two vertical S_8_ rings; Isosurface of the molecular orbits (e) HOMO, (f) LUMO for structure (d). g–j): Theoretical calculation results for N2 nitrogen atom structure. (g) Calculated LUMO and HOMO energy for structure (h). (h) localized structure when a N2 nitrogen atom is inserted between two horizontal S_8_ rings; Isosurface of the molecular orbits (i) HOMO, (j) LUMO for structure (h). Yellow ball: sulfur atom, blue ball: nitrogen atom.

Here, S‐dots were employed to construct deep energy states, leveraging the large bandgap of bulk sulfur (≈2.8 eV),^[^ ^7^ ^]^ which facilitates the formation of such states within the bandgap. Specifically, in contrast to bulk sulfur, the energy levels associated with red‐light emission in S‐dots are in the deep energy state region within the bandgap, thereby serving as a good platform for our study. In addition, as a new class of heavy‐metal‐free quantum dots, S‐dots have garnered significant attention due to their combined merits^[^ ^8^ ^]^ and versatile applications,^[^ ^9^ ^]^ implying their promising potential in photonics. Our first step was to introduce these deep energy states into the bandgap. To achieve this, we carefully considered elements that could generate red light‐associated deep energy levels. Several critical factors were taken into account when selecting an appropriate atom: 1. The electron shell structure of the chosen atom should resemble that of sulfur to facilitate its incorporation into the sulfur crystal structure. 2. The atom should be readily available. 3. The atom should be non‐toxic to ensure compatibility with biomedical applications. After a thorough analysis, we identified nitrogen and oxygen as potential candidates. Recent studies^[^ ^9^, ^10^ ^]^ suggest that oxygen incorporation in S‐dots primarily leads to green light emission. Thus, we opted to introduce nitrogen atoms to generate red light‐associated deep energy states. To further validate the feasibility of nitrogen‐induced deep energy states, we conducted density functional theory (DFT) calculations. As illustrated in **Figure** **1**a, the sulfur crystal consists of S₈ ring structures, making it reasonable to insert a nitrogen atom between two S₈ rings.

After a detailed analysis of the sulfur crystal structure, we identified two possible sites for nitrogen atom insertion: the interval between two vertical S₈ ring structures (N1 in Figure 1b) and the interval between two horizontal S₈ ring structures (N2 in Figure 1b). The calculated energy gap between the Highest Occupied Molecular Orbital (HOMO) and the Lowest Unoccupied Molecular Orbital (LUMO) for the N1 structure is ≈2.02 eV (≈614 nm), placing it within the red spectral range (see Figure 1c,d). Figure 1e,f illustrate the isosurfaces of the HOMO and LUMO states for the N1 structure. Similarly, the HOMO‐LUMO gap for the N2 structure is ≈2.04 eV (≈608 nm), also within the red spectral range (see Figure 1g,h). Figure 1i,j depict the corresponding HOMO and LUMO isosurfaces for the N2 structure. These results indicate that both N1 and N2 structures exhibit similar HOMO and LUMO energy levels, with energy gaps consistently falling within the red spectral range. This confirms the feasibility of nitrogen atom incorporation for generating red light‐associated deep energy states.

Furthermore, it is essential to consider whether the deep energy states reside in the core or on the surface of the S‐dots. Deep energy states typically undergo multiple ionization processes, leading to a broad energy distribution, a low density of states, similar to the defect, with nonradiative recombination. Therefore, manipulation of these deep energy states is necessary to achieve efficient red light emission. If the deep energy states are located within the core of the S‐dots, they are enclosed and isolated from the environment by the surface, making their regulation challenging. In contrast, if these states are situated on the surface, they are directly exposed to the environment, allowing for more effective control and modulation. Based on this analysis, we introduce deep energy states on the surface of the S‐dots by incorporating nitrogen atoms, which facilitate their regulation and enhance red light emission.

**Figure 2:**
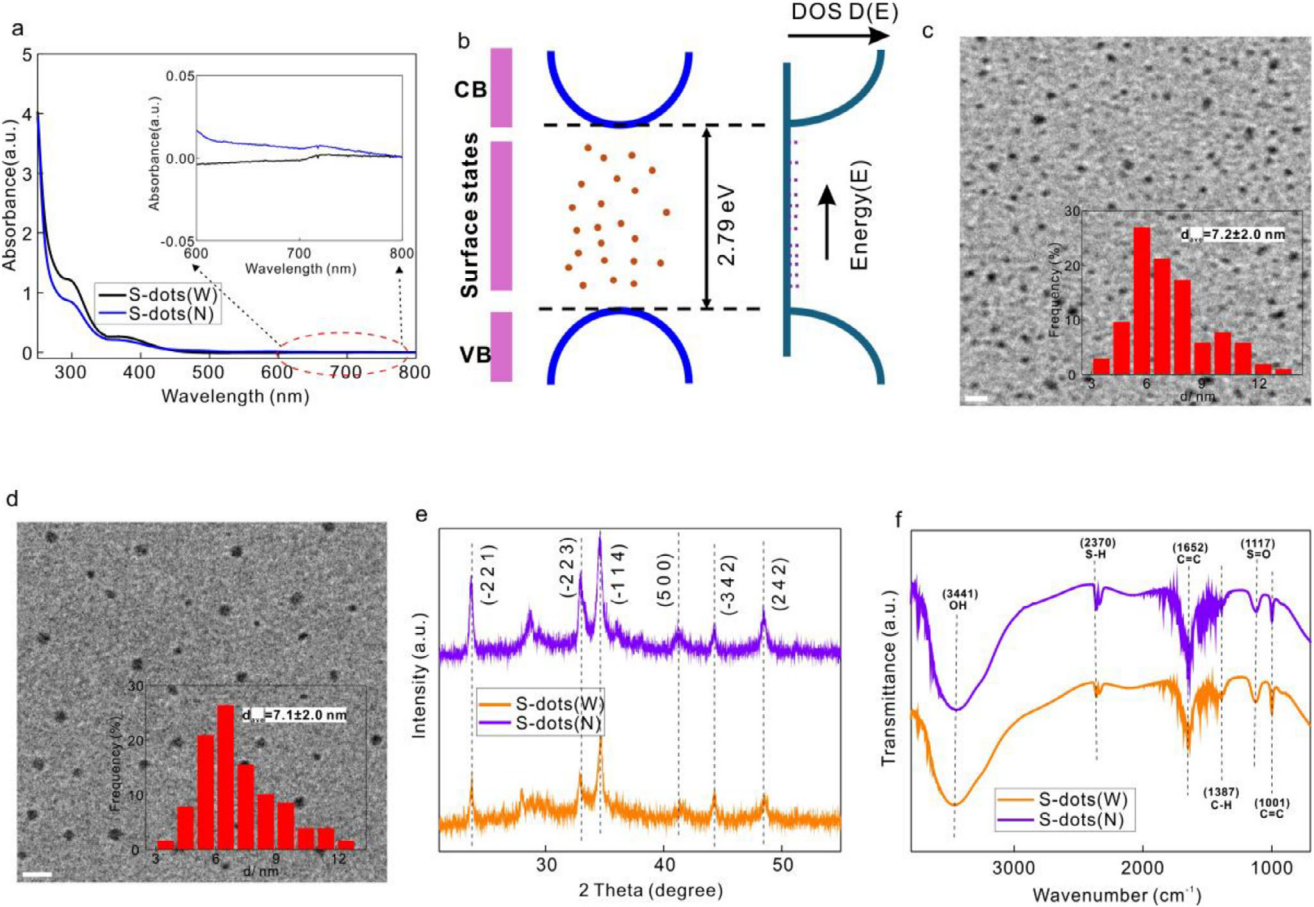
a) Absorption spectra of S‐dots with nitrogen atom incorporation(S‐dots(N)) and without nitrogen atom introduction (S‐dots(W)), and inset is the enlarged absorption spectra in the longer wavelength range. b) Illustration of the electronics structure and density of states (DOS) for S‐dots(N) sample. c) TEM image for S‐dots(W) and inset is the corresponding size distribution. d) TEM image for S‐dots(N) and inset is the corresponding size distribution. e) XRD spectra for S‐dots(W) and S‐dots(N). f) FTIR spectra for S‐dots (W) and S‐dots(N). S‐dots(W): sulfur quantum dots without nitrogen atom. S‐dots(N): sulfur quantum dots with nitrogen atoms. Scale bar for (c) and (d): 20 nm.

We selected a nitrogen‐containing molecule (methylene blue) to generate surface deep energy states via a top‐down hydrothermal approach. Details of the experimental procedure can be found in the Experimental Section. For comparison, we also synthesized sulfur quantum dots without nitrogen incorporation, referred to as S‐dots (W). As shown in **Figure** **2**a, sulfur quantum dots synthesized without nitrogen do not exhibit any detectable red‐light absorption, which is consistent with the large bandgap limitation of bulk sulfur.^[^ ^7^ ^]^ In contrast, upon the introduction of nitrogen atoms (S‐dots(N)), a new weak absorption band emerges in the red spectral range (inset of Figure 2a). The electronic structure of S‐dots(N) is shown in Figure 2b. The multiple ionization processes of the nitrogen atom introduce numerous deep‐level energy states spanning a wide energy range within the band gap of the sulfur core. These deep energy states, characterized by a low density of states typically associated with defect states, contribute to the broad and tiny absorption observed at longer wavelengths in Figure 2a. Transmission electron microscopy (TEM) measurements indicate that S‐dots(W) and S‐dots(N) exhibit a similar size distribution of 7.2 and 7.1 nm, respectively, as illustrated in Figure 2c,d. This similarity rules out quantum confinement as the origin of the newly observed red absorption band. Previous reports^[^ ^11^ ^]^ suggest that quantum confinement effects in S‐dots become significant only when their size is below 5 nm and that such effects typically shift the absorption spectrum toward shorter wavelengths, thus cannot contribute to the red light absorption. This is consistent with our data and analysis.

To further elucidate the mechanism underlying deep energy state generation, we analyzed the crystal structure and surface bonding characteristics of the S‐dots. We suggested that nitrogen atoms are inserted between two S₈ rings on the surface of the S‐dots. Given the top‐down synthesis approach, the primary sulfur crystal structure should remain similar for with and without N atom‐containing S‐dots, as confirmed by X‐ray diffraction (XRD) analysis. The XRD diffraction peaks at 23.6°, 32.9°, 34.6°, 41.5°, 44.3°, and 48.5° imply that S‐dots are predominantly composed of the orthorhombic S₈ phase, with detailed peak assignments provided in Figure 2e. Fourier‐transform infrared spectroscopy (FTIR) measurements (Figure 2f) further reveal that the surface bonding characteristics of S‐dots remain largely unchanged regardless of nitrogen incorporation. The broad absorption band ≈3441 cm^−1^ corresponds to the stretching vibrations of O─H groups, which enhance the water solubility of S‐dots due to their hydrophilic nature. The absorption band near 2370 cm^−1^ is attributed to S─H stretching vibrations. Additionally, the absorption bands at 1652 and 1001 cm^−1^ are associated with the stretching and bending vibrations of C═C, separately. The peaks at 1387 and 1117 cm^−1^ correspond to C─H bending vibrations and S═O stretching vibrations, respectively. These results demonstrate that while nitrogen atoms positioned between two S₈ ring structures contribute to deep energy state formation and the emergence of a new absorption band in the red spectral range, they do not alter the primary crystal structure or surface functional groups of S‐dots.

Deep energy states within the bandgap undergo multiple ionization processes, generating numerous deep energy levels. As a result, these states exhibit a low density of states but are distributed across a broad energy range (Figure 2b), leading to weak yet prolonged absorption at longer wavelengths— a phenomenon we indeed observed. As shown in Figure 2a, a tiny absorption feature appears after 600 nm, further confirming that the new absorption band originates from surface deep energy states. Due to their low density of states and broad energy dispersion, these deep energy states resemble defect states, which typically act as nonradiative recombination centers or exhibit low emission efficiency. Consequently, the photoluminescence quantum yield (PLQY) of the red light emission was measured to be only ≈0.41%. Since the surface deep energy states are introduced via nitrogen atoms on the surface, we further investigated how the amount of the nitrogen source influences deep‐level state formation.

**Figure 3:**
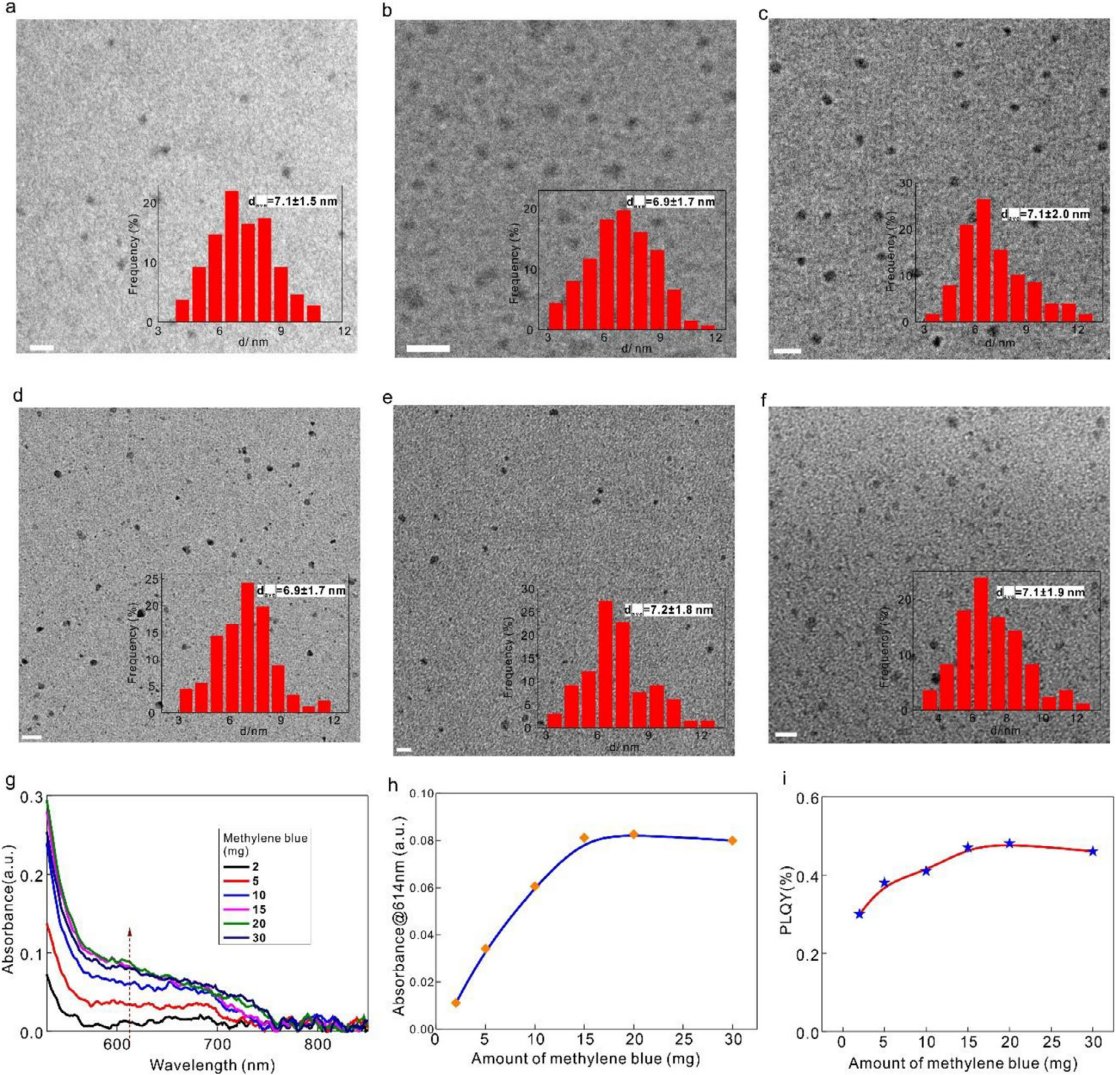
a–f) TEM images of S‐dots(N) synthesized with different amount of methylene blue: (a) 2 mg, (b) 5 mg, (c) 10 mg, (d) 15 mg, (e) 20 mg, (f) 30 mg; insets are the corresponding size distribution. Scale bar: 20 nm. g) Absorption spectra of S‐dots(N) synthesized with different amounts of methylene blue. h) The absorbance changes at 614 nm for absorption spectra in (g). i) Photoluminescence quantum yield (PLQY) of S‐dots(N) when different amounts of methylene blue are utilized.

As shown in Figure **3**a–f, the average size of sulfur quantum dots remains nearly constant despite the increase in nitrogen source concentration. The measured diameter ranges from 6.9 to 7.2 nm as the amount of nitrogen source varies from 0 to 30 mg. However, the absorption spectra exhibit a completely different trend. The absorbance in the red spectral range increases monotonically with the addition of nitrogen source and saturates when the amount exceeds 15 mg, as shown in Figure 3g,h. These observations can be explained as follows: At low nitrogen concentrations (<15 mg), increasing the amount of methylene blue leads to a higher incorporation of nitrogen atoms into the intervals of surface S₈ rings, thereby enhancing red light absorption. However, once all available surface S₈ ring intervals are occupied by nitrogen atoms, further nitrogen addition no longer increases red light absorption. While the absorption continues to rise (when N source < 15 mg) due to the formation of more surface deep energy states, both radiative and nonradiative recombination centers increase simultaneously. As a result, the photoluminescence quantum yield of sulfur quantum dots remains low, showing only a slight increase to 0.48%, even when the nitrogen source concentration is increased a lot, as depicted in Figure 3i.

### 2.2 Surface Ionization Annealing Leads to the Bright Red Light Emission of Sulfur Quantum Dots

**Figure 4:**
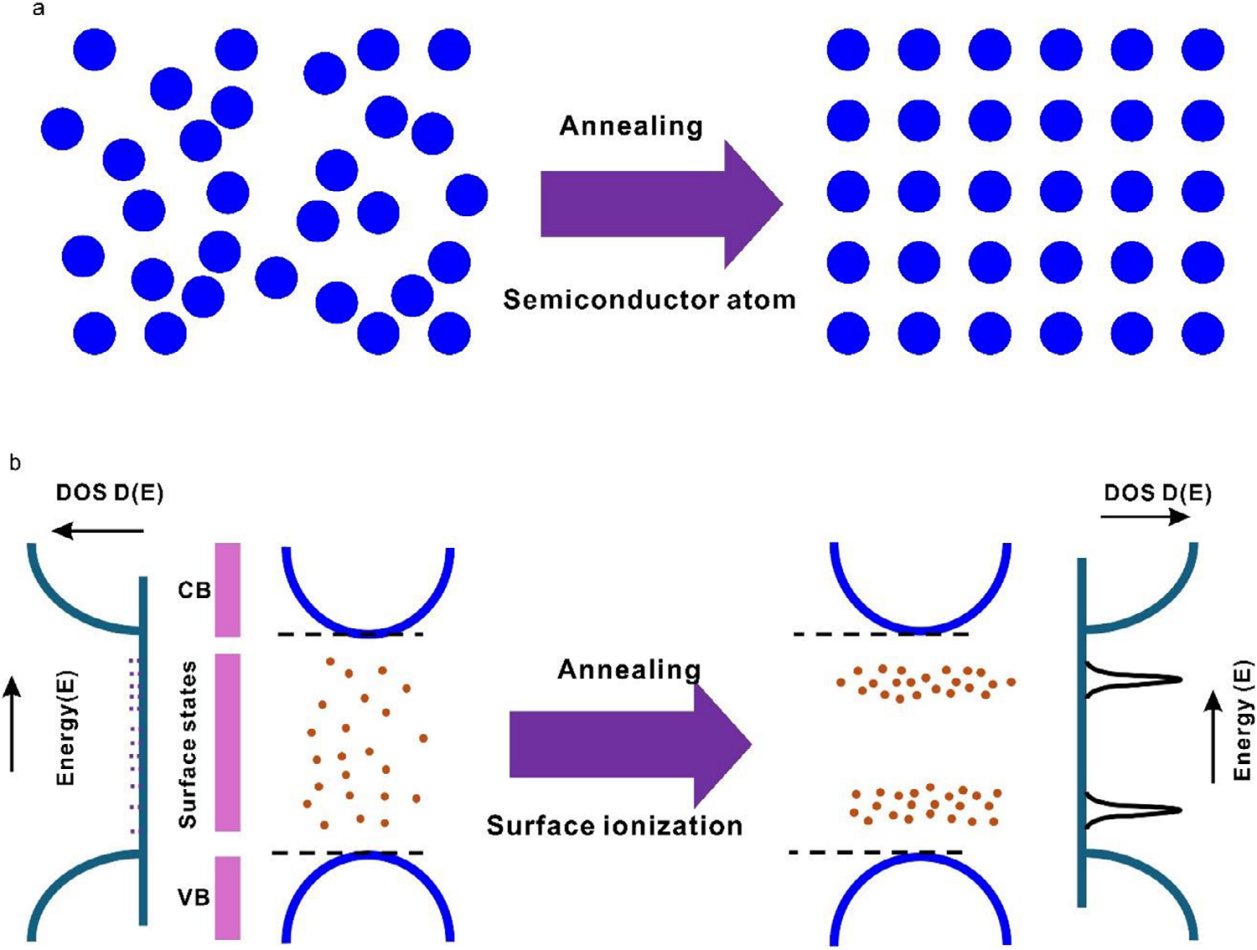
a) Illustration of the annealing process in semiconductors. The randomly distributed atoms are converted into a well‐ordered structure. b) Illustration of surface ionization annealing in sulfur quantum dots. The randomly and broadly distributed, nonradiative surface deep energy levels are converted into “well‐ordered” and “narrow distributed” red light‐emitting energy levels.

Our data suggests that the increase of density of surface deep energy level states is very limited by utilizing more N source, and more importantly, an increased density of these states cannot contribute to an improved photoluminescence quantum yield. As illustrated in Figure 2b, the multiple ionization process of deep‐level states leads to the formation of numerous deep energy levels with a low density of states within the bandgap of S‐dots. This results in weak absorption (Figure 2a) and low PLQY (Figure 3i). Inspired by annealing in semiconductors, which transforms randomly distributed atoms into a well‐ordered structure to reduce defects and enhance performance (see illustration in **Figure** **4**a), we hypothesize that if we can convert randomly and broadly distributed, nonradiative deep energy levels into “well‐ordered” red light‐emitting energy levels. It may significantly increase the density of states of these deep levels, leading to band‐edge‐like absorption (see the depiction in Figure 4b). Since this process resembles annealing but is applied in the context of surface ionization‐related energy levels, we refer to it as “Surface Ionization Annealing”. Additionally, as nonradiative deep energy levels disappear, the PLQY is expected to improve significantly.

**Figure 5:**
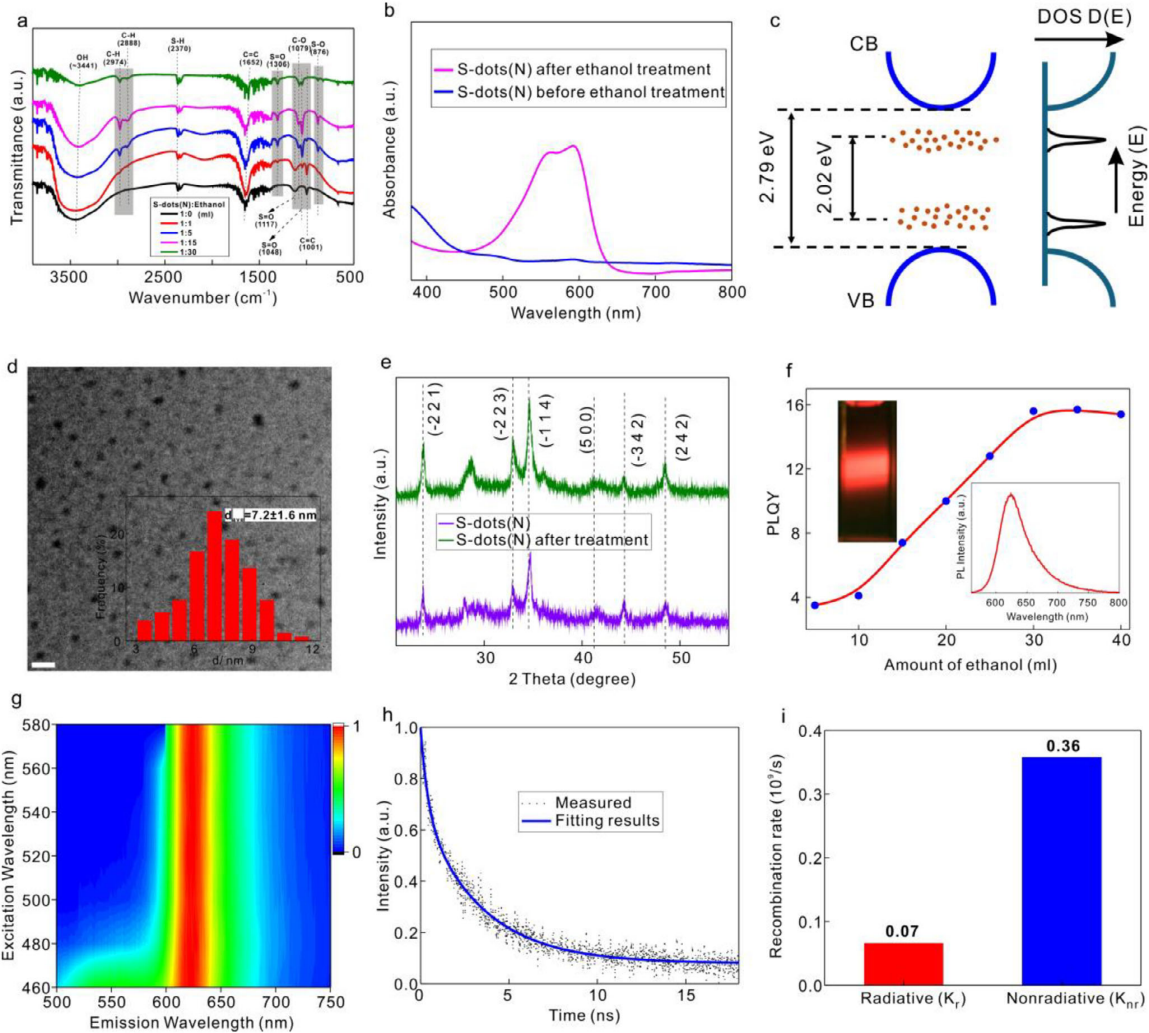
a) FTIR spectra for sulfur quantum dots when different amounts of ethanol are added. b) Absorption spectra of S‐dots(N) before and after ethanol treatment. c) Illustration of the electronics structure and density of states (DOS) for S‐dots(N) after ethanol treatment. d) TEM image for S‐dots(N) with ethanol treatment, and inset is the corresponding size distribution. Scale bar: 20 nm. e) XRD spectra for S‐dots(N) before and after ethanol treatment. f) PLQY of S‐dots(N) when different amounts of ethanol are employed, and insets are the PL spectra and fluorescent image of red emissive S‐dots(N). g) PL spectra of S‐dots(N) with ethanol treatment when the excitation wavelength varied from 460 to 580 nm. h) Time resolved PL spectra of S‐dots(N) after ethanol treatment. i) The radiative and non‐radiative recombination rate for the S‐dots(N) after ethanol treatment.

Since electric potential plays a critical role in deep energy state multiple ionization,^[^ ^12^ ^]^ we regulate this ionization process by controlling surface charge and surface dangling bonds, which are strongly correlated with surface electric potential. This surface state modification can be achieved through ethanol treatment, as ethanol is a strongly polar molecule. The interaction between the polar ethanol molecules and the relatively non‐polar S‐dots structure could significantly alter surface charge and surface dangling bonds. As shown in **Figure** **5** a, FTIR analysis reveals significant modifications in the surface dangling bonds of S‐dots(N) with increasing ethanol concentration. Two new C─H stretching vibrations appear at 2974 and 2888 cm^−1^ in ethanol‐treated S‐dots(N). Additionally, absorption peaks at 1306 and 876 cm^−1^, corresponding to the stretching vibrations of S═O and S─O, gradually appear as the ethanol concentration increases. Concurrently, the S═O stretching vibration (1117 cm^−1^) and C═C bending vibration (1001 cm^−1^) decrease, while the C─O stretching vibration (1079 cm^−1^) and S═O stretching vibration (1048 cm^−1^) appear. Moreover, zeta potential measurements show a decrease from −10.2 to −18.0 mV of S‐dots(N) after ethanol treatment, indicating successful regulation of the surface charge. All of these imply the modification of the surface electric potential. Notably, after ethanol treatment, the absorption spectra of S‐dots(N) undergo a significant transformation, with the emergence of a distinct band‐edge‐like absorption, confirming the substantially increased density of states, as shown in Figure 5b. The corresponding electronic structure and density of states illustration are presented in Figure 5c.

The size and crystal structure of S‐dots(N) after ethanol treatment were also analyzed to rule out structural changes as the origin of the observed band‐edge‐like absorption. Figure 5d shows that the average size of the S‐dots(N) after ethanol treatment remains ≈7.2 nm, which is similar to that of untreated S‐dots(N) (Figure 2d). Additionally, XRD measurements reveal that the crystal structure remains unchanged before and after ethanol treatment, with all diffraction peaks corresponding to the orthorhombic S₈ phase, as presented in Figure 5e. These results confirm that neither size alternation nor crystal structure modification contributes to the band‐edge‐like absorption. Instead, this phenomenon arises from surface ionization annealing, which induces a high density of states. Furthermore, the elimination of nonradiative deep‐level states leads to a dramatic enhancement in PLQY, which reaches 15.6% when an excess amount of ethanol is used (Figure 5f). This process closely resembles annealing in semiconductors, where ionized deep energy states are “reorganized” into a more “structured form” (red light emitting deep energy levels). Thus, we term this method as surface ionization annealing: annealing surface‐ionized deep energy states.

Moreover, it is important to emphasize that our proposed mechanism differs fundamentally from traditional surface passivation strategies.^[^ ^13^ ^]^ While surface passivation can suppress nonradiative recombination states and enhance PLQY, it does not alter the absorption spectra. In contrast, our surface ionization annealing not only improves PLQY but also introduces band‐edge‐like absorption, highlighting its distinct and unique nature. Since both the high absorption and PLQY are necessary for the bright light emission, our mechanism provides an alternative strategy for bright light emission realization. The composition of the S‐dots(N) after ethanol treatment was analyzed using X‐ray photoelectron spectroscopy (XPS), with the results presented in Figure S1 (Supporting Information). Our findings confirm that S‐dots(N) primarily consist of atomic sulfur, along with a significant presence of sulfite and sulfonyl/sulfonate groups, similar to a previous report.^[^ ^8a^ ^]^ Our data also indicates that nitrogen atoms do not significantly affect the oxygenated surface states of S‐dots(N), and the detailed analysis is shown in SI Figure S2. The element composition of S‐dots(N) is determined as follows: S:28.8%, O: 24.5%, N 13.6%, C:25.4%, Na: 5.3%, Cl: 2.4%. Furthermore, a comprehensive analysis presented in Figure S3 (Supporting Information) uncovers that neither methylene blue nor its interaction with S‐dots (W) contributes to the absorption or bright red‐light emission. This confirms that the new absorption band and red‐light emissive properties are intrinsic to the fabricated S‐dots alone. Additionally, we evaluated different types of nitrogen sources for the generation and regulation of deep energy states within the band gap of S‐dots, and the results are presented in Figures S4 and S5 (Supporting Information). There are no obvious deep energy states in S‐dots(W), and the absorption is primarily from the band edge transition of S‐dots. As a result, after ethanol treatment, the absorption remains dominated by the band edge transition of S‐dots, as shown in Figure S6 (Supporting Information).

The emergence of band‐edge‐like absorption results in remarkably stable red light emission spectra, even when the excitation wavelength varies from 460 to 580 nm, as presented in Figure 5g. This behavior differs from previously reported S‐dots emissions, where the emission wavelength shifts with changes in the excitation wavelength.^[^ ^9^, ^14^ ^]^ These observations are fully consistent with the characteristics of band‐edge‐like absorption. Additionally, the lifetime of the red light emission was determined to be 2.36 ns, as shown in Figure 5h. The radiative and nonradiative recombination rates were also calculated to be 0.07 *10^9^/s and 0.36 *10^9^/s, respectively, as shown in Figure 5i. The comparison for the S‐dots(N) samples before and after ethanol treatment is shown in Figure S7 and Table S1 (Supporting Information).

### 2.3 Toxicity Evaluation and In Vivo Bio Imaging

**Figure 6:**
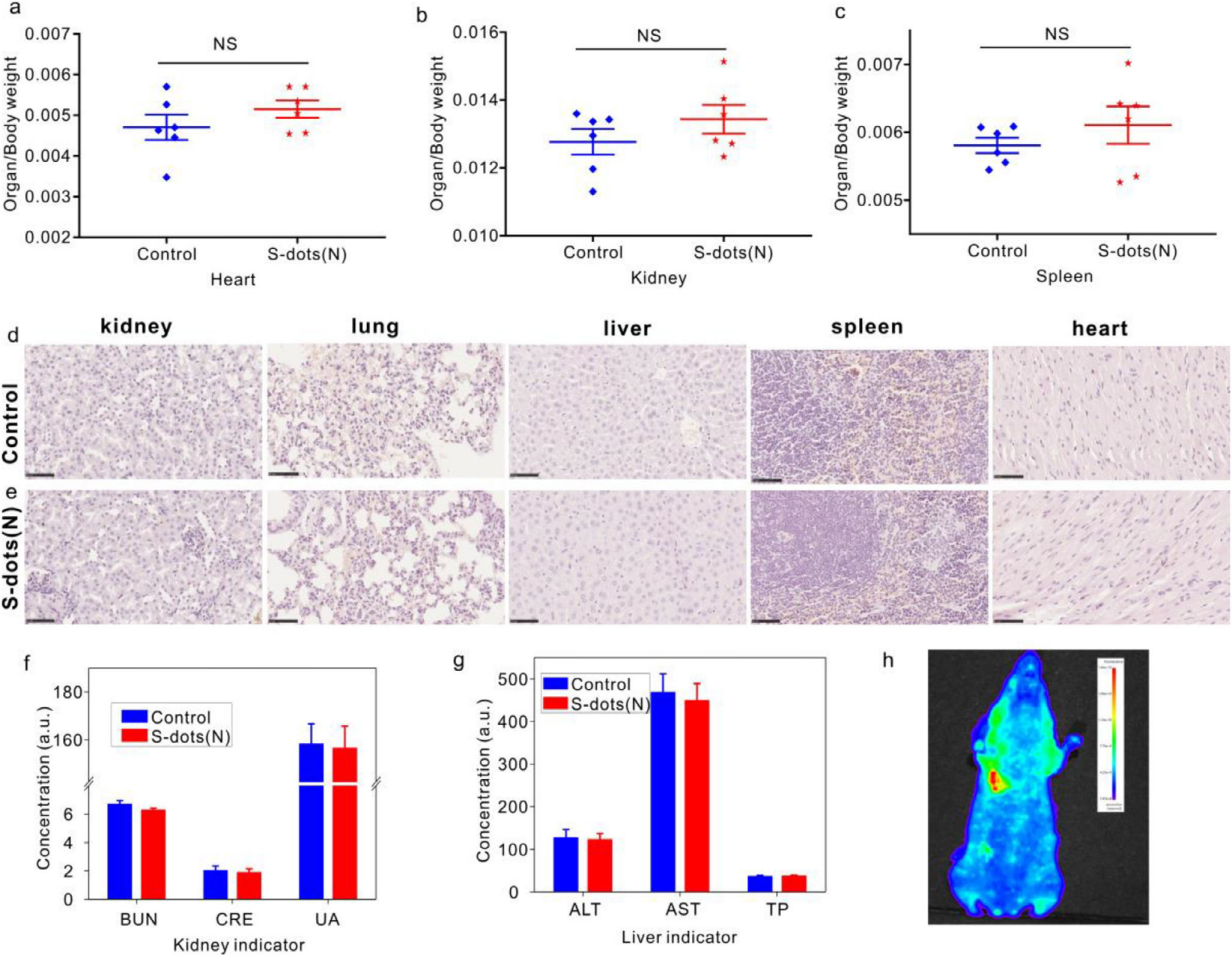
a–c) Organ weight index (organ weight/mice body) of (a) heart, (b) kidney, and (c) spleen for control and S‐dots(N) exposure group. d,e): Histological analysis (H&E) of kidney, lung, liver, spleen and heart for control (d) and S‐dots(N) treatment group (e). Scale bar: 50 µm. Blood biochemistry analysis for kidney f) and liver g). BUN: urea nitrogen, CRE: creatinine, UA: uric acid, ALT: alanine transaminase, AST: aspartate aminotransferase, TP: total protein. Fluorescence h) image of mouse after 3 h of S‐dots(N) injection. For a–c: n = 6, for f–g: n = 3. For plots a–c, f–g: Error bars show mean ± SEM and the one‐side Student's T‐test was used to calculate significance. ^*^ *p* < 0.05, ^**^ *p* ≤ 0.01, ^***^ *p* ≤ 0.001, ^****^ *p* ≤ 0.0001. ns: no significant difference.

The bright red light emission of S‐dots(N) enables in vivo bioimaging applications. Comprehensive toxicity assessment is indispensable and crucial for materials to be applied in the biomedical field. The in vitro cytotoxicity of S‐dots(N) was evaluated using the MTT assay. As shown in Figure S8a,b (Supporting Information), S‐dots(N) did not induce significant cell death in 4T1 and EMT6 cells after 48 h of co‐culture at a concentration of 100 µg mL^−1^. Furthermore, we conducted an in vivo toxicity assessment using two experimental groups: a control group and an S‐dots(N) treatment group. Detailed information is provided in the Experimental Section. The comparable body weights of mice in the control and S‐dot(N) treatment groups (Figure S8c, Supporting Information) suggest that S‐dots(N) do not affect overall growth. Additionally, the organ weight index (organ weight/body weight) showed no significant differences between the two groups, indicating that S‐dots(N) do not cause severe impairment to major organs (**Figure** **6**a–c for the heart, kidney, and spleen; Figure S8d–e (Supporting Information) for the liver and lungs). Besides, histopathological staining was performed to evaluate potential histological or microstructural changes in major organ tissues following S‐dots(N) exposure. The organs, including the kidney, lung, liver, spleen, and heart, were fixed, sectioned, and stained with hematoxylin and eosin (H&E) prior to microscopic examination. Representative microscopic images are shown in Figure 6d,e. No significant structural impairment or abnormalities were observed in any of the examined organs after S‐dot(N) exposure. Given the crucial role of the kidney and liver in the in vivo clearance of nanomaterials, we performed biochemical analysis to evaluate the functional impact of S‐dot(N) exposure on these organs. No significant differences were observed between the S‐dot(N) exposure and control groups in kidney markers (blood urea nitrogen (BUN), creatinine (CRE), and uric acid (UA), Figure 6f) or liver markers (alanine transaminase (ALT), aspartate aminotransferase (AST), and total protein (TP), Figure 6g). These results suggest that S‐dot(N) intake does not cause obvious impairment of kidney or liver function. Collectively, our in vitro and in vivo assessments reveal that the red‐emissive S‐dots(N) exhibit excellent biocompatibility, supporting their suitability for in vivo applications. Leveraging the high PLQY of red‐emitting S‐dots(N), we demonstrated in vivo bioimaging. Mice were intravenously injected with S‐dots(N) (10 mg kg^−1^) and imaged using an in vivo Xtreme system (Bruker Preclinical) with an excitation wavelength of 535 nm. The fluorescence image of the intravenously injected mice displays clear PL originating from the S‐dots. Bright fluorescence could be observed near the liver after 3 h of post‐injection, as presented in Figure 6h.

## 3 Conclusion

In this work, we realize the bright light emission from deep energy states. By proposing a new deep energy state regulation mechanism, termed “surface ionization annealing”, we realize a high density of states and band‐edge‐like absorption (thus strong absorption) from deep energy states. Moreover, our regulation strategy simultaneously achieves a high photoluminescence quantum yield (PLQY) from deep energy states. Consequently, the simultaneous realization of a high density of states (high absorption) and high PLQY enables bright light emission from deep energy states. Our results represent the first realization of red light emission from sulfur quantum dots.

Over the past few years, significant progress has been made in the photoluminescent properties of sulfur quantum dots, with blue, green, and even ultraviolet light emissions achieving quantum yields surpassing 85%,^[^ ^15^ ^]^ 15%,^[^ ^10^ ^]^ and 20%,^[^ ^11^ ^]^ respectively. However, the large band gap of bulk sulfur (≈2.8 eV, ≈440 nm) makes achieving red light emission challenging.^[^ ^11^ ^]^ Compared to green or blue light emission, red light emission exhibits reduced photo‐damage and autofluorescence in animal tissues, making it better for in vivo biomedical applications. Furthermore, red light emission is crucial for the development of full‐color emitting devices, such as white light‐emitting diodes. Hence, currently, the applications and explorations of sulfur quantum dots are largely hindered, although they are recognized to be endowed with distinct advantages such as heavy metal‐free nature, earth abundance, water solubility, and superior antimicrobial behavior.^[^ ^16^ ^]^ Thus, the realization of bright red light emission not only paves the way for S‐dot‐based biomedical applications, such as biosensing, phototheranostics, and in vivo imaging etc., but also opens up opportunities for S‐dot‐based optoelectronics, including white light emission.

Additionally, bright light emissions in inorganic semiconductors have traditionally been attributed to band‐edge transitions or shallow energy states, implying that the emission energy is confined by the band gap. In contrast, we demonstrate that deep energy states, which are far from the band‐edge energy, can still exhibit strong absorption and bright light emission. Our results offer new insights and an alternative approach for achieving bright light emissions from semiconductors.

## 4 Experimental Section

### Ethics

Female Balb/c mice aged 6–8 weeks were obtained from Gene Line Bioscience, Inc. All experiments involving mice were conducted in compliance with the regulations set forth by the China Committee for Research and Animal Ethics (SYXK 2022‐0015).

### Material Synthesis–Sulfur Quantum Dots Synthesis

All of the chemicals and reagents for the sulfur quantum dots synthesis were purchased from Sigma–Aldrich and used without further purification. S‐dots (W) synthesis: Briefly, 0.3 g sulfur, 0.8 g NaOH, 0.1 mL PEG (polyethylene glycol) 400 were dissolved into 10 mL water and transferred into a Teflon‐line autoclave chamber and reacted at 160 °C for 4 h. S‐dots(N) synthesis: 0.3 g sulfur, 0.8 g NaOH, 0.1 mL PEG (polyethylene glycol) 400 and different amount of methylene blue (2, 5, 10, 15, 20 and 30 mg) were dissolved into 10 mL water and transferred into a Teflon‐line autoclave chamber and reacted at 160 °C for 4 h.

### Material Synthesis–Ethanol treatment

Different amounts of ethanol (CAS: 64‐17‐5; >99.8%, analytical reagent grade) were added into the raw S‐dots aqueous solution with vigorous stir for another 3 h. Supernatant was collected and purified using 0.45 µm pore size filters for three times to remove the large particles. For bio‐assays, the S‐dots solution was dry and re‐dissolved into water and purified with the dialysis bag (MWCO: 12K Da) (D6066, Sigma; Cellulose tubing; Avg. flat width 35 mm (1.4 in), MWCO 12 000 Da; retains proteins with a molecular weight >12 000 Da.).

### Optical Characterization

For photoluminescence (PL) spectra, measurements were performed using a Fluorescence Spectrophotometer (Varian Cary Eclipse) with a xenon flash lamp as the excitation source. The scan speed was set to medium, with a data interval of 1 nm. Both the excitation and emission bandwidths were 3 nm. The absorption spectra were measured using a Cary‐100 UV–vis spectrometer (Varian) or a homemade measurement system. The UV–vis scan rate was set to 600 nm min^−1^, with a data interval of 1 nm. For time‐resolved measurements, PL spectra were obtained using a streak camera (Optronis) coupled with a 300 mm monochromator. A 400 nm femtosecond laser was used as the excitation source. The speed is set to 330 ps mm^−1^, and the MCP is set to 850 V.

### MTT Assay for the Cytotoxicity Evaluation of Sulfur Quantum Dots

In a 96‐well plate, 100 µL of medium with an initial cell count of ≈8000 cells was cultured. Six different concentrations of S‐dots: 0 µg mL^−1^ (as a reference), 12.5, 25, 50, and 100 µg mL^−1^ were used to examine the viability of both cell lines. After incubation in a 5% CO2 incubator at 37 °C for 24 h, the aqueous S‐dots solution at different concentrations was added to the medium and continued the treatment for an additional 24 or 48 h. Subsequently, the medium was removed, treated the cells with 50 µL of 0.1% MTT solution for 30 min. After removing the MTT reagent, 100 µL of isopropyl alcohol was added and shook it for 10 min. Finally, cell viability was assessed by measuring the optical density at 490 nm. Three replicate wells were conducted for each concentration.

### In Vivo Toxicity Evaluation and Bio Imaging

Healthy female Balb/c mice aged 6–8 weeks were randomly assigned to two groups: the PBS (control) group and the S‐dots(N) injected group. The mice received intraperitoneally injections of either 10 mg kg^−1^ of S‐dots(N) or PBS buffer every other day and were observed for a period of 30 days. During the observation period, the mice's weights were measured every three days. On the 31st day, the mice were humanely euthanized for further evaluation. The heart, kidney, liver, lung, and spleen were harvested, weighed, and blood samples were collected. All animal experiments were conducted in accordance with ethical guidelines established in China. The collected organs were fixed using 4% neutral buffered formalin, embedded in paraffin, sectioned at 4 µm thickness, and stained with hematoxylin and eosin (H&E). The resulting slides were examined using digital microscopy with a 40× objective lens. A histological analysis of the organs was performed to assess whether the sulfur quantum dots had induced any tissue damage, lesions, or other pathological effects. All experiments involving mice were conducted in compliance with the regulations set forth by the China Committee for Research and Animal Ethics.

For in vivo imaging, the mice were injected intravenously with S‐dots (N) (10 mg kg^−1^) and imaged in an in vivo Xtreme system (Bruker Preclinical) with an excitation wavelength of 535 nm.

### Statistical Analysis

One‐tailed distribution and two‐sample unequal variance Student's T test were utilized for the evaluation of the differences between two individual groups. P < 0.05 was regarded as statistically significant. ^*^ *p* < 0.05, ^**^ *p* ≤ 0.01, ^***^ *p* ≤ 0.001, ^****^ *p* ≤ 0.0001. ns: no significant difference.

## Conflict of Interest

The authors declare no conflict of interest.

## Supporting information



Supporting Information
