# Supplementary material for: Bright Light Emission from Deep Energy States
Source: Adv Sci (Weinh). 2025 Aug 12;12(41):e09549. doi: 10.1002/advs.202509549 (PMC12591114; doi:10.1002/advs.202509549)
Supplement: Supplementary file 1 — Supporting Information [file ADVS-12-e09549-s001.docx]

**Bright light emission from deep energy states**

Lian Xiao^1 2^*, Sihang Liu^3^, Zhan Yu^4^, Yong Yi^5^, Rui Duan^6^, Quanchao Du^7^, Yugang Zhao^8^, Xuehong Zhou^2^, Van Duong Ta^9^, Edwin Kok Lee Yeow^7^, Yi Huang^3^*, Zhi-Gang Zheng^1^*, Handong Sun^6^*

*Emails: [lxiao003@e.ntu.edu.sg](mailto:lxiao003@e.ntu.edu.sg), [huangyi_buaa@buaa.edu.cn](mailto:huangyi_buaa@buaa.edu.cn), [zgzheng@ecust.edu.cn](mailto:zgzheng@ecust.edu.cn), [hdsun@um.edu.mo](mailto:hdsun@um.edu.mo)

^1^ School of Physics, East China University of Science and Technology, Shanghai 200237, China

^2^ Division of Physics and Applied Physics, School of Physical and Mathematical Sciences, Nanyang Technological University, 21 Nanyang Link, Singapore 637371

^3^ Research Institute of Aero-Engine, Beihang University, No.37 XueYuan Road, Haidian District, Beijing, China, 100083

^4^ Beijing An Zhen Hospital, Affiliated of Capital University of Medical Sciences

^5^ Center of Growth, Metabolism and Aging, Key Laboratory of Bio-Resource and Eco-Environment of Ministry of Education, College of Life Sciences, Sichuan University, 610064, Chengdu, China

^6^ Institute of Applied Physics and Materials Engineering, University of Macau, Macao SAR 999078, China

^7^ School of Chemistry, Chemical Engineering & Biotechnology, Nanyang Technological University, Singapore, 637371, Singapore

^8^Shanghai Key Laboratory of Multiphase Flow and Heat Transfer in Power Engineering, School of Energy and Power Engineering, University of Shanghai for Science and Technology, Shanghai 200093, China

^9^ Department of Optical Devices, Le Quy Don Technical University, Hanoi, 100000, Vietnam

**Key words**: sulfur quantum dots, red light emission, deep energy states, density of states, bio imaging.


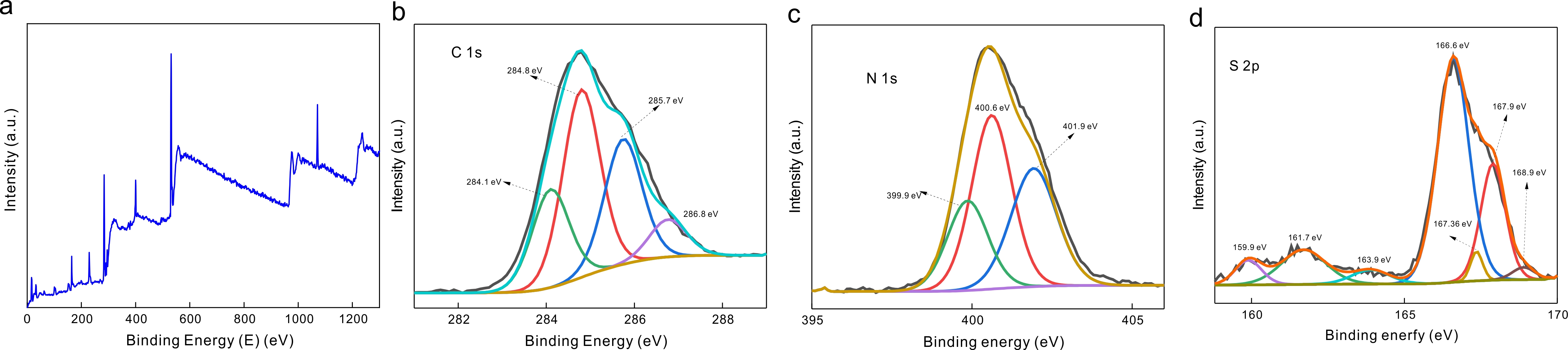


**Figure S1** XPS spectra of S-dots(N). (a) Full XPS scan. (b) High resolution of C 1s. (c) High resolution XPS spectra of N 1s. (d) High resolution XPS spectra of S2p.

The full XPS scan (Figure S1a), along with the deconvoluted N1s, C1s and S2p spectra. The C1s spectra (Figure S1b) can be split into four peaks located at 284.1 eV, 284.8 eV, 285.7 eV, and 286.8 eV, corresponding to the C=C, C-O, C-N, and C=N groups, respectively. The deconvoluted N1s spectra (Figure S1c) implies the presence of pyridinic (399.9 eV), pyrrolic (400.6 eV), and oxidized (401.9 eV) nitrogen compositions.

The high-resolution **XPS spectra of S 2p (Figure S1d)** exhibits seven distinct peaks. Peaks at **159.9 eV, 161.7 eV, and 163.9 eV** correspond to **atomic sulfur**, while binding energy peaks at **166.6 eV, 167.36 eV, 167.9 eV, and 168.9 eV** are attributed to **SO₂⁻ (2p₃/₂), SO₂²⁻ (2p₁/₂), SO₃²⁻ (2p₃/₂), and SO₃²⁻ (2p₁/₂),** respectively. These findings confirm that **S-dots(N)** primarily consist of **atomic sulfur**, along with a significant presence of **sulfite and sulfonyl/sulfonate groups**.


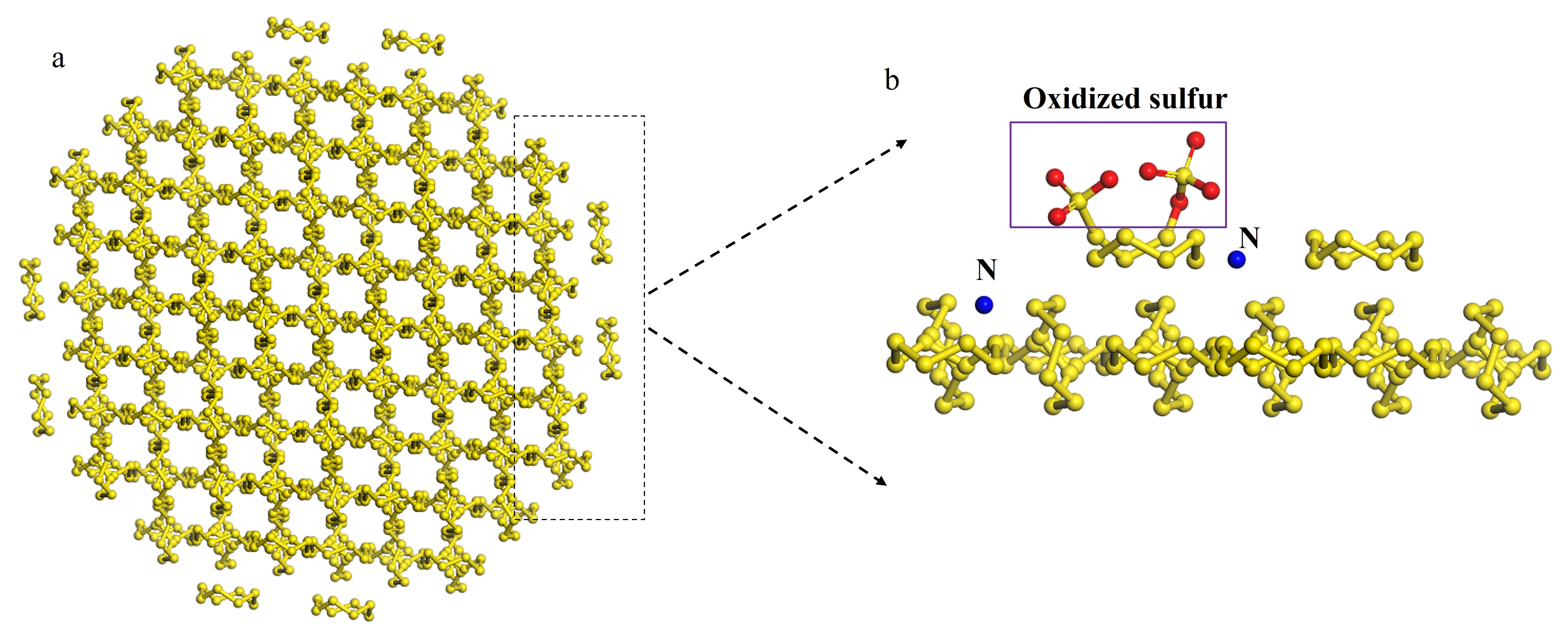


**Figure S2** Illustration of the N atom and oxidization on the S-dots surface. Yellow ball: sulfur atom; blue ball: nitrogen atom; red ball: oxygen atom.

We suggest that nitrogen doping does not reduce the oxygenated surface states of S-dots significantly. The N atom is inserted between two S_8_ rings, as illustrated in the Figure S2b. On the other hand, oxidation of S-dots occurs on the surface sulfur atoms, as depicted in the Figure S2b. Thus, nitrogen atom does not affect the oxidization process obviously. Additionally, the obtained S-dots exhibit a significant portion of oxidized sulfur composition (see Figure S1d), which further indicates that nitrogen atom does not significantly affect the oxygenated surface states of S-dots.


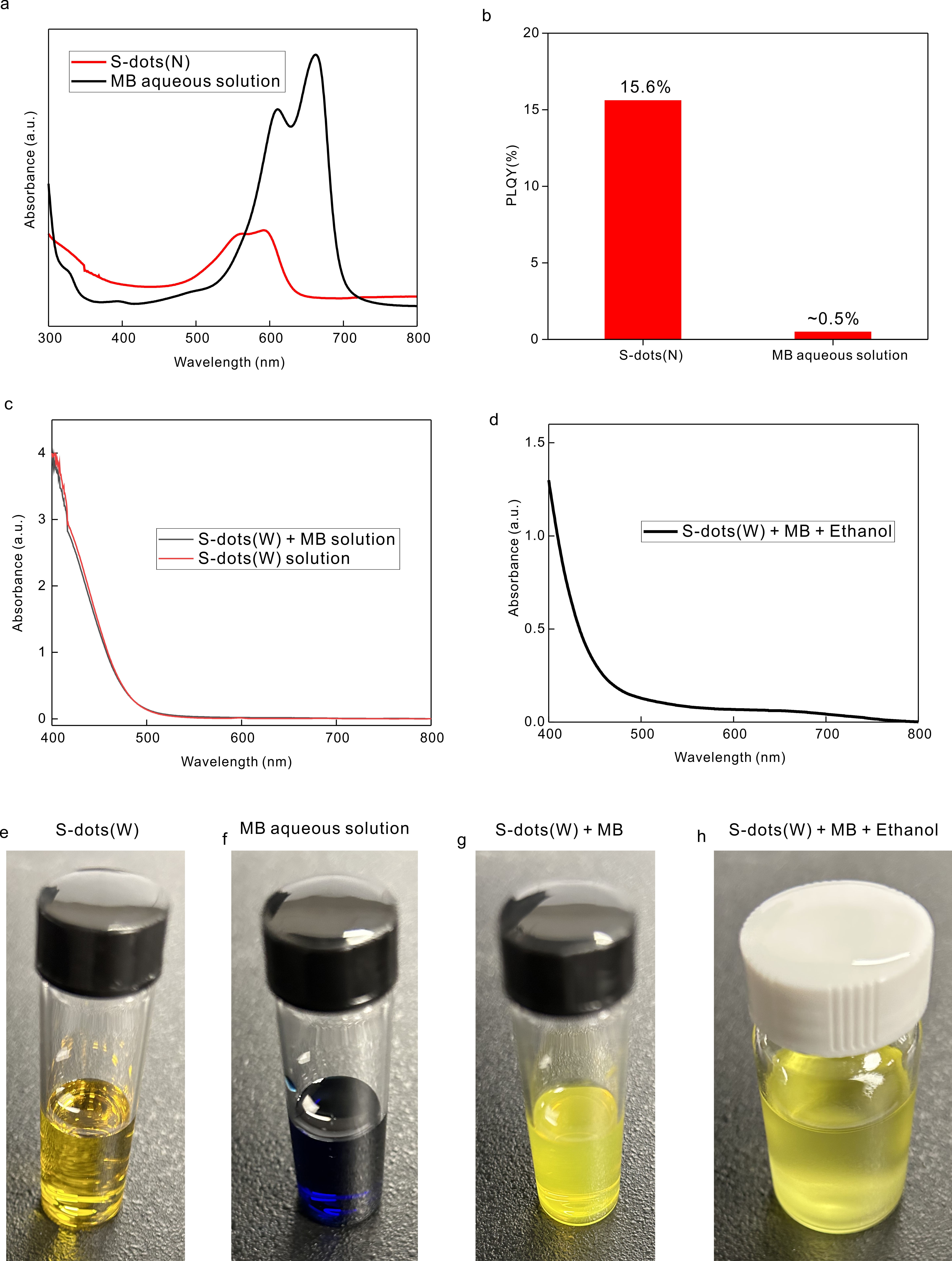


**Figure S3** (a) Absorption spectra of methylene blue (MB) and S-dots(N) aqueous solution. (b) The PLQY of methylene blue and S-dots(N) aqueous solution (c) The absorption spectra of S-dots (W) and the mixture of S-dots (W) and methylene blue. (d) The absorption spectra of the mixture of S-dots (W), methylene blue and ethanol solution. The photograph of the (e) S-dots (W) aqueous solution, (f) methylene blue aqueous solution, (g) the mixture of S-dots (W) aqueous solution and methylene blue, (h) the mixture of S-dots (W) aqueous solution, methylene blue and ethanol solution.

Here, we clarify that the observed red light emission originates from our sulfur quantum dots sample and not from the raw material, methylene blue. As depicted in Figure S3a, the absorption spectra of methylene blue in aqueous solution differ significantly from those of our sulfur quantum dots sample. Furthermore, the photoluminescence quantum yield (PLQY) of methylene blue in aqueous solution is merely around 0.5%, which is considerably lower than the PLQY of our sulfur quantum dots sample, approximately 15.6%.

Additionally, our data unequivocally demonstrates that the interaction between the surface of S-dots (W) (devoid of methylene blue) and methylene blue in an aqueous solution does not result in the absorption and emission of red light. On the contrary, sulfur quantum dots synthesized without the use of methylene blue tend to bleach methylene blue, as evidenced in Figure S3c. Moreover, the introduction of additional ethanol solution does not induce red light emission either (Figure S3d). Images of S-dots (W) (synthesized without the use of methylene blue), methylene blue aqueous solution, the mixture of S-dots (W) and methylene blue aqueous solution, as well as the mixture of S-dots (W), methylene blue aqueous solution, and ethanol are presented in Figure S3e-h. It is evident that the S-dots (W) solution leads to the bleaching of methylene blue. In summary, we can confidently conclude that the red light absorption and emission indeed originate from our sulfur quantum dots sample and not from methylene blue.

**Evaluation of the different nitrogen source**

We tested three types of nitrogen sources (methylene blue, Toluidine Blue O (C_15_H_16_ClN_3_S), and urea (CO(NH₂)₂)) and found that methylene blue is the most efficient nitrogen source for generating deep energy states and bright red light emission. The detailed experimental results are as follows:

Differ from methylene blue, urea (S-dots(urea)) fails to generate deep energy states in S-dots, as evidenced by the absence of absorption spectra in the longer wavelength range (Figure S4a). In contrast, Toluidine Blue O(S-dots(TBO)) performs similarly to methylene blue. It can generate obvious deep energy states within the S-dots band gap, as indicated by appearance of absorption in the longer wavelength range, as shown in Figure S4b. After ethanol treatment, the deep energy states regulation mechanism, “surface ionization annealing”, occur, leading to the band edge like absorption and bright red light emission, as presented in Figure S5.

Although both methylene blue and Toluidine Blue O can generate deep energy states within the band gap of S-dots, the methylene blue-treated sample(S-dots(N)) exhibits a higher PLQY(15.6%) compared to S-dots(TBO)(PLQY 12.4%) after ethanol treatment. Consequently, we chose methylene blue as the nitrogen source.


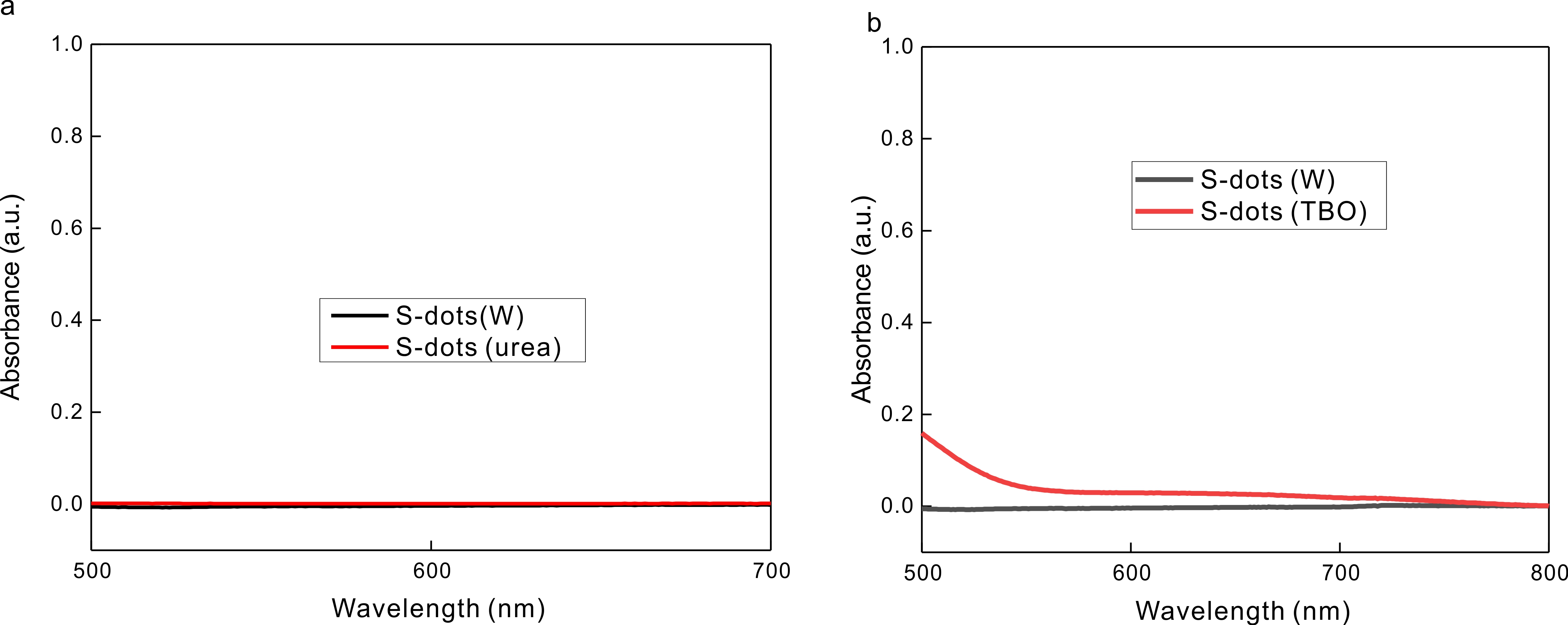


**Figure S4** (a) Absorption spectra of S-dots(W) and S-dots(urea) in longer wavelength range. (b) Absorption spectra of S-dots(W) and S-dots (TBO) in longer wavelength range. S-dots(W): sulfur quantum dots synthesized without utilizing N source. S-dots(urea): sulfur quantum dots synthesized using urea as the N source. S-dots(TBO): sulfur quantum dots synthesized using toluidine blue O as the N source.


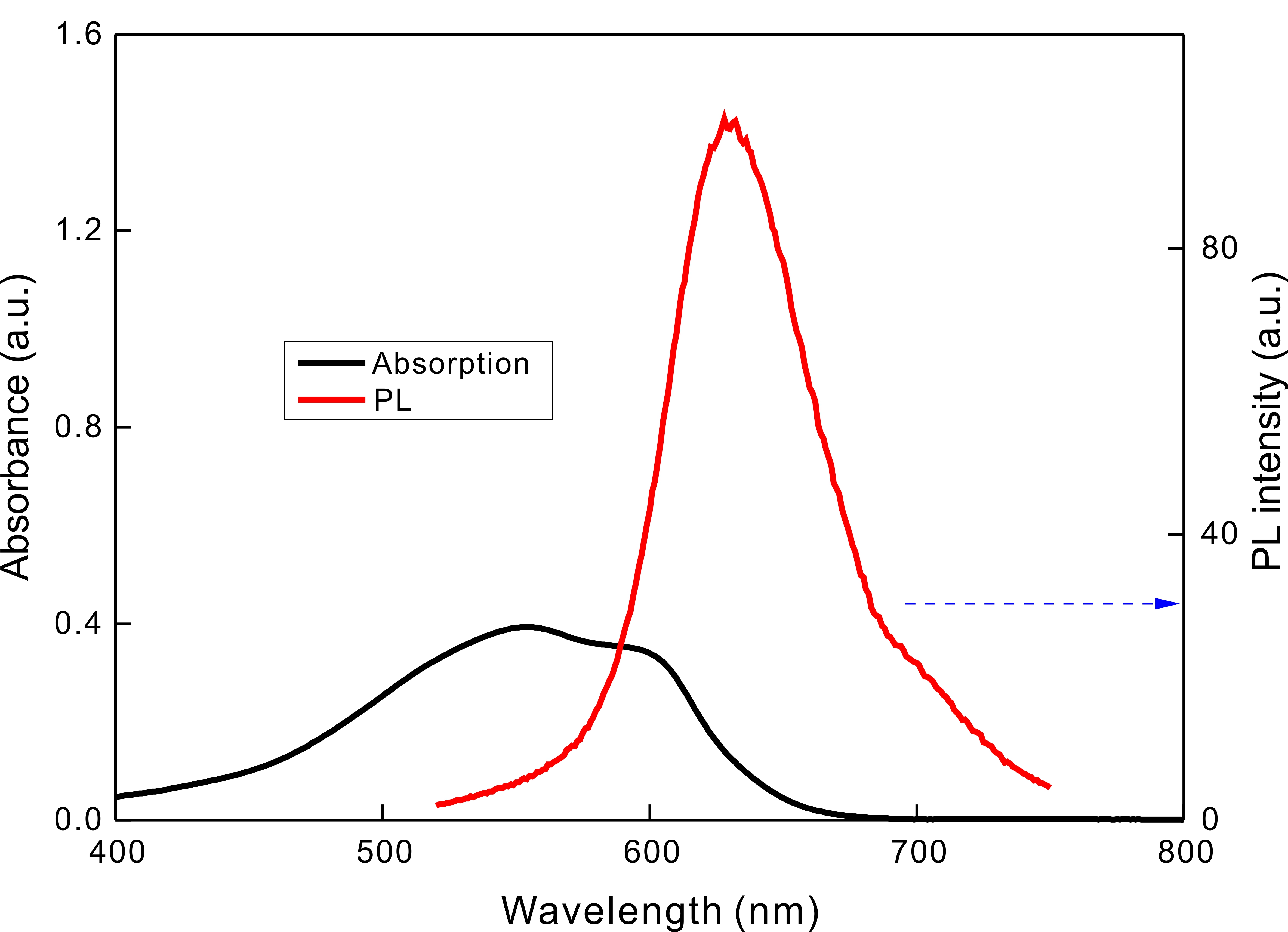


**Figure S5** Absorption and PL spectra of S-dots (TBO) after ethanol treatment. PL spectra excitation wavelength: 500 nm.


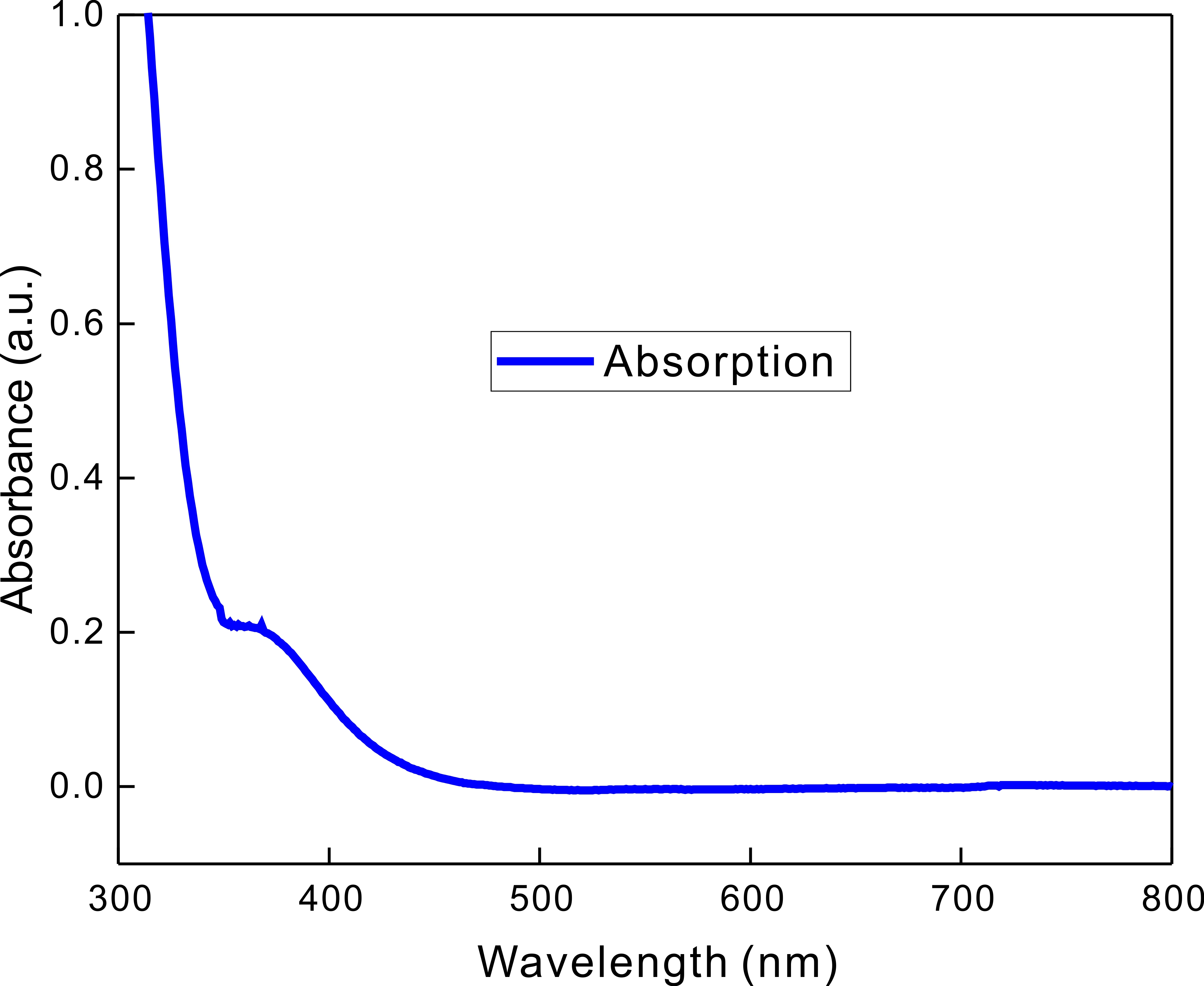


**Figure S6** The absorption spectra of S-dots(W) after ethanol treatment.

**The time-resolved photoluminescence spectra**

The time-resolved photoluminescence (TRPL) spectra for S-dots(N) samples before and after ethanol treatment are shown in Figure S7a and Table S1. As observed, the lifetime of S-dots(N) before ethanol treatment is shorter than that of S-dots(N) after ethanol treatment, which implies a faster loss of hot carriers in the excited states. Moreover, the radiative and non-radiative recombination rates for S-dots(N) before and after ethanol treatment are presented in Figure S7b. It can be clearly seen that ethanol treatment results in a significant increase in the radiative recombination rate, from 0.003 * 10^9^/s to 0.07 *10^9^/s, accompanied by a decrease in the non-radiative recombination rate, from 0.07 * 10^9^/s to 0.36 * 10^9^/s.

**Table S 1** Fitting results of time resolved decay curves.

| Sample | $\tau_{1}$ (ns) | $\tau_{2}$ (ns) | $D_{1}$ | $D_{2}$ | $R_{1}$ | $R_{2}$ | $\tau_{ave}$ (ns) |
| --- | --- | --- | --- | --- | --- | --- | --- |
| After ethanol treatment | 0.41 | 3.37 | 0.31 | 0.61 | 0.34 | 0.66 | 2.36 |
| Before ethanol treatment | 0.35 | 2.43 | 0.49 | 0.50 | 0.49 | 0.51 | 1.41 |

We use the double exponential function$I(\mathrm{t})=\mathrm{I}_{0}+D_{1}\bullet e^{-t/\tau_{1}}+D_{2}\bullet e^{-t/\tau_{2}}$to fit the PL decay curve of S-dots, where R_i_ is the relative ratio, determined as $R_{i}=D_{i}/\sum_{i} D_{i}$and $\tau_{ave}$ is the average PL lifetime, defined as$\tau_{ave}=\sum_{i} R_{i}\tau_{i}$. The fitting data for different emission wavelength are presented in Table S1.


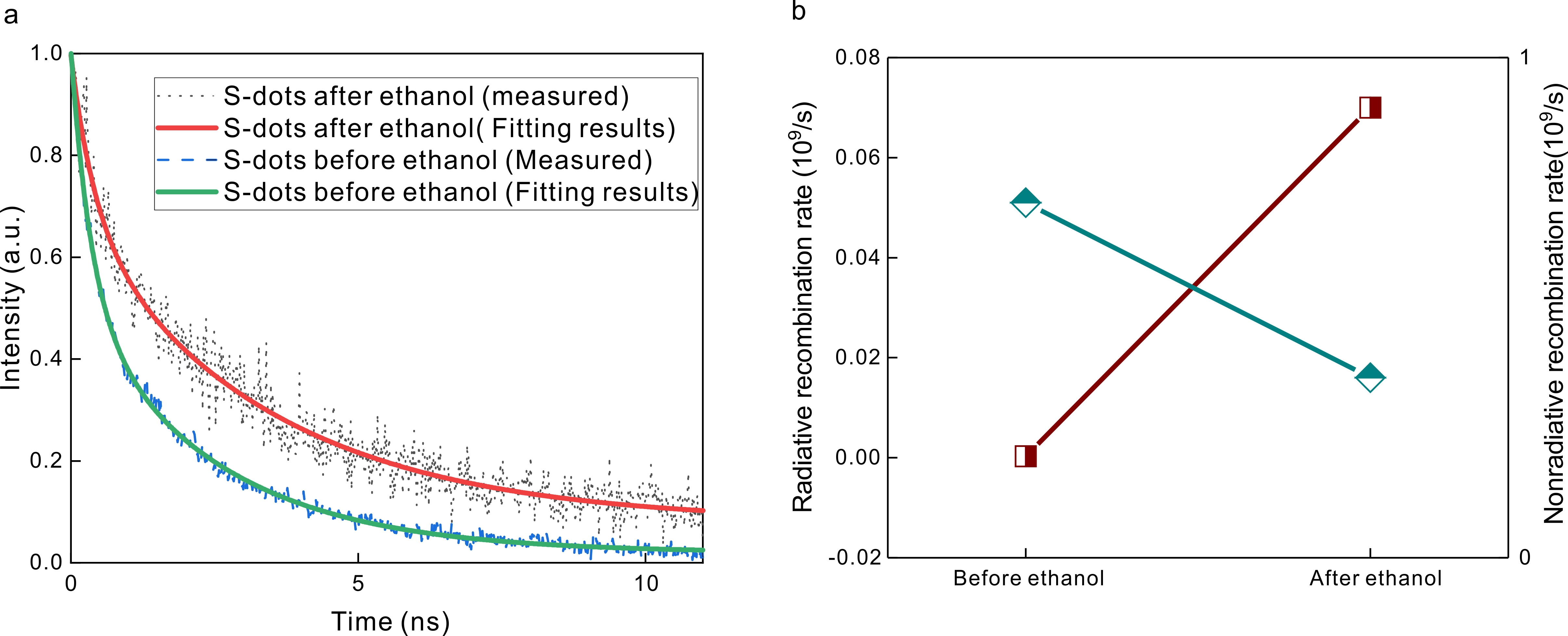


**Figure S7** (a) Time resolved PL spectra for S-dots(N) before and after ethanol treatment. (b) The radiative and non-radiative recombination rate for S-dots(N) before and after ethanol treatment.


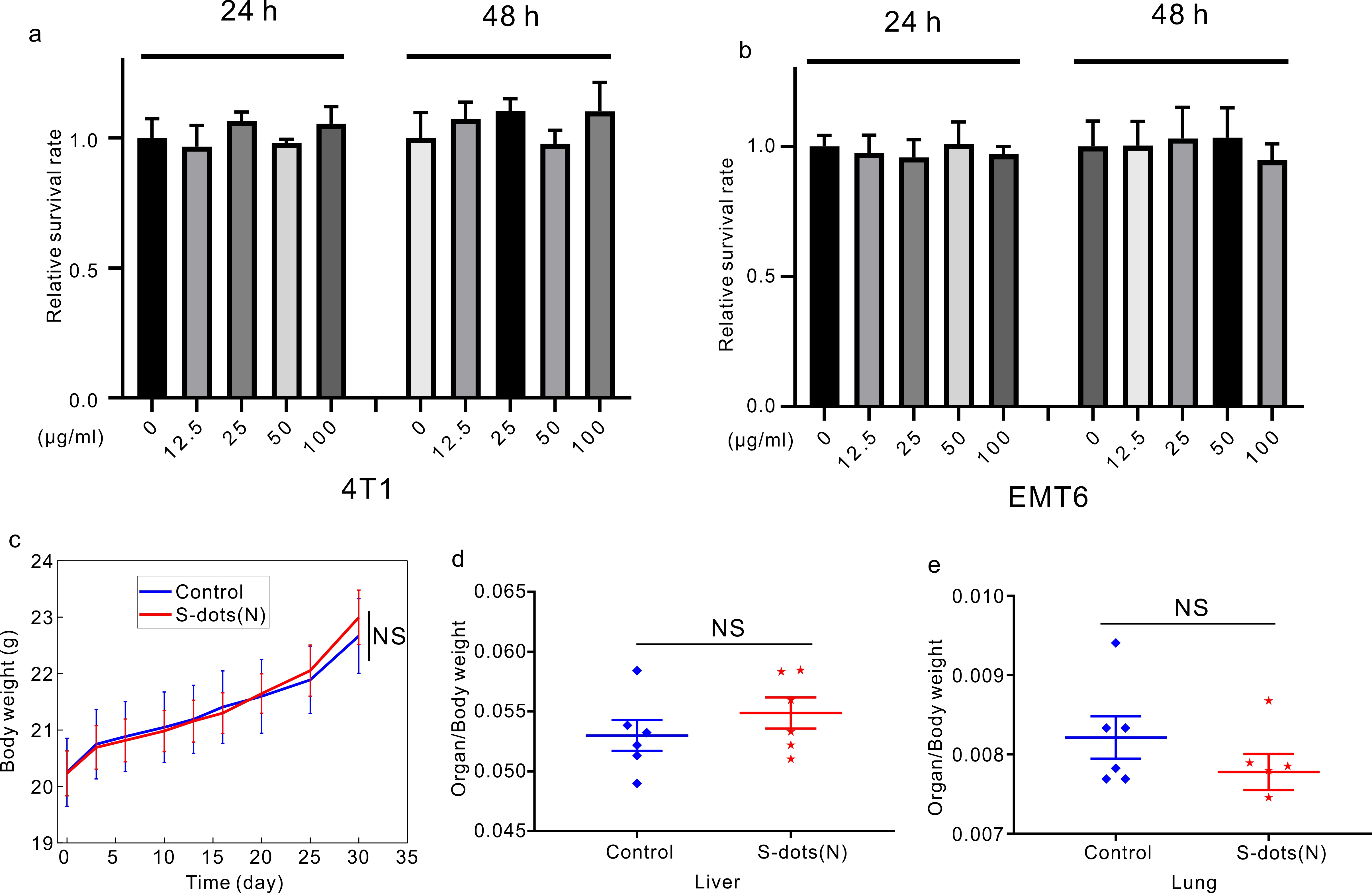


**Figure S8** Cell viability when incubated with S-dots(N), (a) 4T1, (b) EMT6. (c) The mice body weight for control and S-dots(N) treatment group. (d) – (e) organ weight index of liver and lung. For a-b: n= 3 or 4, for c-e: n=6. For plots a-e: Error bars show mean ± SEM and the one-side Student's T-test was used to calculate significance. *P < 0.05, **P ≤ 0.01, ***P ≤ 0.001, ****P ≤ 0.0001. ns: no significant difference.
